# Endocannabinoid System-Related Inflammation and Progression of Autosomal Dominant Polycystic Kidney Disease

**DOI:** 10.3390/ijms27094087

**Published:** 2026-05-02

**Authors:** Paulina Simankowicz, Barbara Dołęgowska, Małgorzata Marchelek-Myśliwiec, Katarzyna Dołęgowska, Jacek Różański, Joanna Stępniewska

**Affiliations:** 1 Department of Nephrology, Transplantology and Internal Medicine, Pomeranian Medical University, Powstancow Wielkopolskich 72, 70-111 Szczecin, Poland; berenika777@gmail.com (P.S.); malgorzata.marchelek@gmail.com (M.M.-M.); jacek.rozanski@pum.edu.pl (J.R.); 2 Department of Microbiology, Immunology and Laboratory Medicine, Pomeranian Medical University, Powstancow Wielkopolskich 72, 70-111 Szczecin, Poland; barbara.dolegowska@pum.edu.pl (B.D.); katarzyna.dolegowska@pum.edu.pl (K.D.)

**Keywords:** polycystic kidney disease, chronic kidney disease, endocannabinoid system

## Abstract

The endocannabinoid system is a complex regulatory network whose functioning is associated with maintaining homeostasis and regulating immune response. The aim of this study was to evaluate the relationship between endocannabinoid system activity, inflammation, and the progression of chronic kidney disease (CKD) in patients with autosomal dominant polycystic kidney disease (ADPKD). The study included 105 participants: 60 individuals with ADPKD and 45 healthy volunteers. From a single venous blood draw, concentrations of anandamide (AEA), 2-arachidonoylglycerol (2-AG), tumor necrosis factor α (TNF-α), and interleukin 6 (IL-6) were measured in EDTA plasma. Basic laboratory parameters were also assessed, including complete blood count, iron metabolism indices, electrolyte panel, and azotemia parameters. There were statistically significant differences in the concentrations of both endocannabinoids, with higher mean values observed in the control group (*p* < 0.001). IL-6 concentrations were significantly higher in the ADPKD group compared with controls (*p* < 0.001). Although TNF-α concentrations were higher in the ADPKD group than in the control group, these differences did not reach statistical significance. Statistically significant correlations were also identified between inflammatory markers, endocannabinoid concentrations, and indices of renal function. Patients with ADPKD exhibit an altered endocannabinoid profile characterized by reduced AEA and 2-AG concentrations, which correlates with disease progression and poorer kidney function. The endocannabinoid system may modulate inflammation in ADPKD.

## 1. Introduction

The endocannabinoid system is a complex cell signaling network that plays a key role in maintaining organismal homeostasis and is responsible for numerous functions, including the regulation of hormone levels, body temperature, sleep, mood, appetite and many others [1]. The endocannabinoid system comprises cannabinoid receptors (CB1 and CB2), endogenous agonists, and enzymes that regulate the synthesis and degradation of the system’s endogenous ligands [2,3]. The best-known endogenous agonists of the endocannabinoid system are anandamide (AEA) and 2-arachidonoylglycerol (2-AG) [1,4].

The primary synthesis enzymes are N-acyl-phosphaditylethanolamine-specific phospholipase D (NAPE-PLD) for AEA and diacylglycerol lipases (DAGLs) for 2-AG. The basic metabolic enzymes are fatty acid amide hydrolase (FAAH), which metabolizes AEA, and monoacylglycerol lipase (MAGL), which metabolizes 2-AG [5]. These enzymes therefore play a crucial role in maintaining endocannabinoid homeostasis, whereby the levels of different endocannabinoids are kept in balance. Moreover, by modulating biosynthetic enzyme activity or inhibiting metabolic enzymes, one can regulate endocannabinoid concentrations and receptor activity [6]. The most important genes associated with the endocannabinoid system include the *FAAH1*, *FAAH2*, and *CNR1* genes. The *FAAH1* and *FAAH2* genes encode enzymes from the fatty acid amide hydrolase family, which are responsible for the degradation of endocannabinoids. The *FAAH1* gene is located on chromosome 1, and its highest expression has been demonstrated in the central nervous system. Inhibition of the *FAAH1* gene contributes to an increase in anandamide levels, which exerts analgesic and anti-inflammatory effects [7]. The *FAAH2* gene is located on the X chromosome. Its expression has been confirmed in many tissues, including the brain. Mutations in the *FAAH2* gene have been linked to lipid metabolism disorders, anxiety disorders, intellectual disability, and autism [8]. The *CNR1* gene is located on chromosome 6 and encodes the cannabinoid receptor CB1. Polymorphisms of the *CNR1* gene are associated with neuropsychiatric disorders such as attention-deficit hyperactivity disorder (ADHD) or post-traumatic stress disorder (PTSD) [9].

Cannabinoid receptors differ in their tissue distribution. CB1 receptors are located primarily in the central nervous system, but also in peripheral tissues such as skeletal muscle, adipose tissue, and the liver. CB2 receptors are found predominantly on cells of the immune system [2,3,10]. This differential distribution underlies distinct functional roles: activation of CB1 is chiefly linked to metabolism, appetite regulation, feeding motivation, and nociception, whereas activation of CB2 modulates immune responses, exerts anti-inflammatory effects, and may attenuate inflammatory pain [11]. Disruption of endocannabinoid homeostasis can contribute to the development of numerous pathological processes [10,12]. Kidney diseases—including autosomal dominant polycystic kidney disease—provide an example in which the endocannabinoid system may participate in regulating glucose homeostasis and other metabolic processes, as well as the chronic inflammatory response that drives disease progression [13,14].

Polycystic kidney disease (PKD) is a genetically determined disorder characterized by numerous cysts in the renal cortex and medulla. The autosomal dominant form manifests in adulthood and is the most common monogenic kidney disease, with a frequency of 1 in 400–1000 live births, accounting for 8–15% of end-stage renal disease cases [15]. The disease is caused by mutations in two main genes, PKD1 and PKD2, which encode polycystin-1 and polycystin-2, respectively [16]. In the OMIM (Online Mendelian Inheritance in Man) database, mutations in these genes are listed under entries #173900 for PKD1 and #613095 for PKD2. The form of the disease associated with PKD1 mutations is characterized by a more severe clinical course and an earlier onset of end-stage renal failure. Conversely, the form related to PKD2 mutations is typically associated with a milder course and a later onset of end-stage renal failure. Over 100 distinct PKD1 mutations account for 85% of cases, while over 80 PKD2 mutations are responsible for the remaining 15% [17]. Furthermore, there are seven minor loci that are generally associated with milder forms of kidney disease. The disease progression linked to the DNAJB11 and ALG5 genes is similar to PKD2. Mutations in the GANAB, ALG8, and PKHD1 genes can present as ADPKD or autosomal dominant polycystic liver disease. A mutation in the PKHD1 gene is also associated with autosomal recessive polycystic kidney disease, which manifests as early as the fetal period or immediately after birth [18]. The age at diagnosis, disease severity, and presence of extrarenal complications depend on the mutation type. Normal renal function is typically preserved until 30–40 years of age, followed by a rapid decline between 40 and 70 years; up to 50% of patients require renal replacement therapy before age 60. As the disease progresses and kidney function deteriorates, disturbances in acid–base balance, calcium phosphate and carbohydrate metabolism, and anemia emerge. Among multiple contributing factors, these processes may also be related to the endocannabinoid system and chronic inflammation. Histologic analyses of kidneys from patients with ADPKD demonstrate interstitial inflammation and fibrosis [19,20]. Available evidence suggests that interstitial inflammation is driven largely by proinflammatory cytokines, including tumor necrosis factor α [21,22,23]. TNF-α and other proinflammatory mediators have been isolated from cyst fluid and urine of patients with ADPKD. Numerous studies further describe the accumulation of inflammatory cells—such as macrophages and T lymphocytes—in the renal interstitium and urine of patients with ADPKD [21,24,25], with macrophages likely playing a central role in cyst progression [26,27,28].

TNF-α and IL-6 are key proinflammatory cytokines that regulate immune responses and inflammation. Elevated levels indicate an ongoing inflammatory process, including in infections, malignancies, and autoimmune diseases. TNF-α is a glycoprotein that exerts its effects by binding to receptors on the cell membrane; two receptor types have been identified (TNFR1 and TNFR2). These receptors are expressed on immunocompetent cells, and their activation stimulates cytokine production and release. TNF-α is produced primarily by activated monocytes and macrophages, and to a lesser extent by other tissues. It is cytotoxic to many tumor cell lines, stimulates hepatic synthesis of acute-phase proteins, increases insulin resistance in peripheral tissues, attracts neutrophils, and promotes phagocytosis.

Interleukin-6 is one of the most important and highly pleiotropic cytokines. It is secreted mainly by monocytes and macrophages in response to interleukin 1 and other proinflammatory cytokines. IL-6 promotes differentiation of B lymphocytes, activates T lymphocytes, stimulates hematopoiesis, and participates in feedback inhibition of TNF production. In addition, it is a pyrogenic factor and stimulates the production of acute-phase proteins.

IL-6 is implicated in the progression of chronic kidney disease [29]. Moreover, monitoring IL-6 levels can be used to assess the risk of cardiovascular disease and cardiovascular mortality in patients with CKD [30]. In the study by Kamińska et al., both IL-6 and TNF-α levels were significantly higher in advanced CKD stages; however, only IL-6 correlated significantly with 5-year mortality risk [31].

The aim of the study was to analyze the concentrations of the two principal endocannabinoids—anandamide and 2-arachidonoylglycerol—in individuals with autosomal dominant polycystic kidney disease at CKD stages 3–5, compared with healthy controls. Furthermore, the results were related to inflammatory status and correlated with two key proinflammatory cytokines, IL-6 and TNF-α.

## 2. Results

### 2.1. The Biochemical and Blood Morphology Parameters

Between-group analyses revealed statistically significant differences in mean creatinine (*p* < 0.001), uric acid (*p* = 0.018), urea (*p* < 0.001), and vitamin D (*p* = 0.003), with higher mean levels in the ADPKD group. Statistically significant differences were also observed for eGFR (estimated Glomerular Filtration Rate) (*p* < 0.001), hemoglobin (HGB) (*p* = 0.026), and red blood cell count (RBC) (*p* = 0.018), with higher mean values in the control group. Kidney enlargement, expressed as increased longitudinal dimensions of both the right and left kidneys in the ADPKD group compared with healthy controls, was typical for ADPKD and was statistically significant.

Two iron-related proteins that are also acute-phase reactants—transferrin (a negative acute-phase protein) and ferritin (a positive acute-phase protein)—were compared between groups. There was a statistically significant difference in transferrin (*p* = 0.008), with higher mean levels in the control group. No statistically significant difference was observed for ferritin. No other significant differences were noted in the remaining biochemical parameters (Table 1).

### 2.2. Endocannabinoid, TNF-α and Interleukin-6 Concentrations

**Figure 1:**
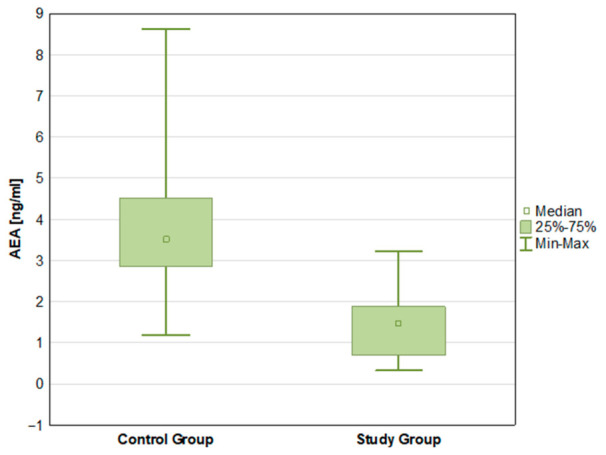
AEA levels in control group and study group.

**Figure 2:**
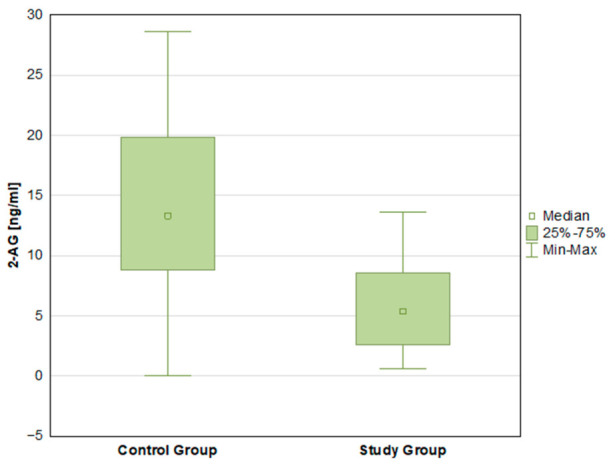
2-AG levels in control group and study group.

**Figure 3:**
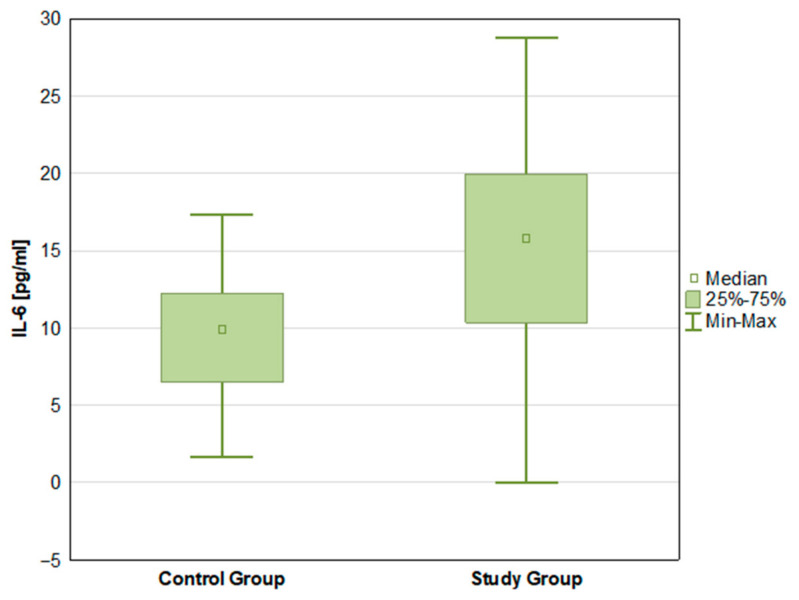
IL-6 levels in control group and study group.

There were statistically significant differences in the mean concentrations of both endocannabinoids—AEA (*p* < 0.001) and 2-AG (*p* < 0.001)—with higher mean values in the control group (Figure 1 and Figure 2). IL-6 concentrations differed significantly between groups (*p* < 0.001), with higher mean levels in the ADPKD group compared with controls (Figure 3). Mean TNF-α concentrations were higher in the ADPKD group than in the control group; however, this difference did not reach statistical significance (Table 2).

### 2.3. Spearman’s Correlations

The Spearman rank correlation coefficient values between endocannabinoid concentrations and age, biochemical and morphological parameters in the study and control groups are presented in Table S1 (Supplementary Materials). Statistically significant relationships (*p* < 0.05) between the concentrations of selected laboratory parameters and the concentrations of AEA and 2-AG are marked in bold.

In the ADPKD group, AEA concentrations showed a positive correlation with transferrin and eGFR and a negative correlation with ferritin, parathyroid hormone (PTH), azotemia indicators (creatinine, urea, uric acid), and age. 2-AG concentrations showed a positive correlation with IL-6 and eGFR and a negative correlation with PTH and azotemia indicators (creatinine, urea, uric acid).

In the control group, a significant negative correlation was observed between AEA concentrations and serum ionized calcium concentrations. 2-AG correlated positively with ferritin, hemoglobin, urea, and IL-6 and negatively with transferrin.

A significant positive correlation was observed between the concentrations of both endocannabinoids in the ADPKD group. No other statistically significant correlations were identified (*p* > 0.05).

Similarly, Spearman correlation coefficients between IL-6 and TNF-α concentrations and age, biochemical, and morphometric parameters in both groups are presented in Table S2 (Supplementary Materials). Statistically significant associations (*p* < 0.05) between selected parameters and inflammatory markers (IL-6 and TNF-α) are indicated in bold.

In the ADPKD group, IL-6 correlated positively with PTH, vitamin D3, 2-AG, and right and left kidney length. In the control group, IL-6 correlated positively with urea, ferritin, 2-AG, hemoglobin, and right kidney length, and negatively with transferrin.

In the ADPKD group, a positive correlation was found between TNF-α concentrations and vitamin D3 and transferrin, whereas in the control group, an inverse correlation was observed only with serum ionized calcium concentration. No other statistically significant correlations were found (*p* > 0.05).

**Figure 4:**
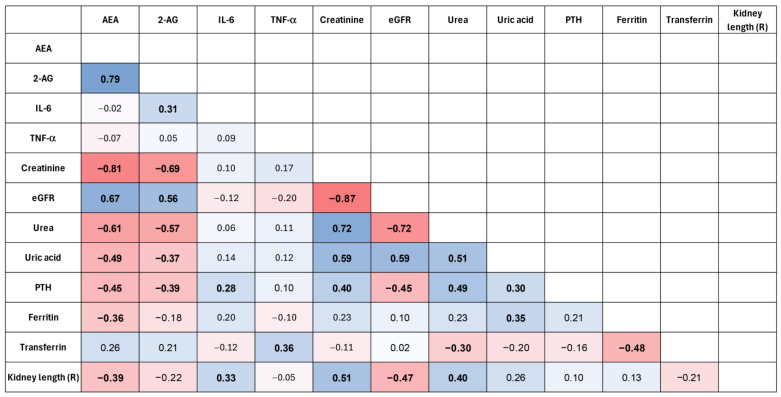
Spearman correlation heatmap showing relationships between endocannabinoids (AEA, 2-AG), inflammatory markers (IL-6, TNF-α), and selected clinical parameters in the ADPKD group. Statistically significant correlations (*p* < 0.05) are shown in bold. The colors in the heatmap represent the strenght and direction of the Spearman corelation coefficient. Values close to +1 indicate a strong positive correlation (red color), values close to; −1 indicate a strong negative correlation (blue color), and values around 0 indicate little or no correlation (neutral colors).

**Figure 5:**
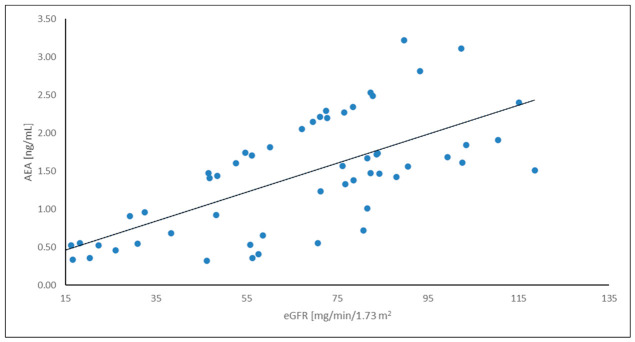
Relationship between AEA concentration and eGFR in the ADPKD group.

**Figure 6:**
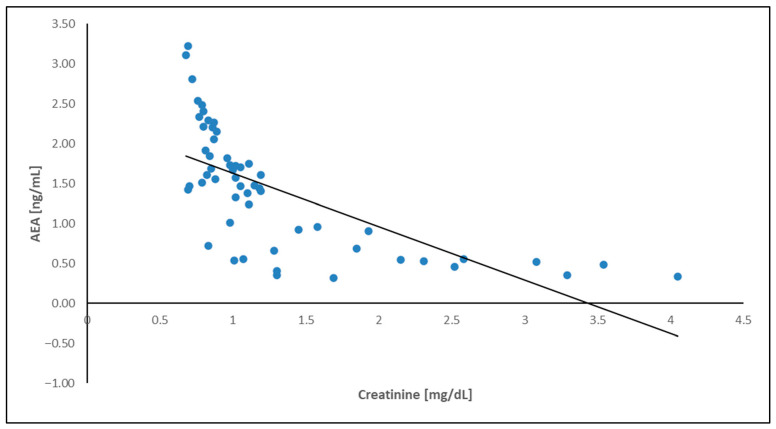
Relationship between AEA concentration and serum creatinine in the ADPKD group.

**Figure 7:**
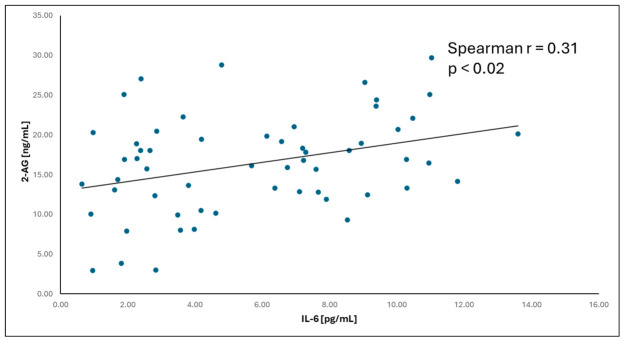
Relationship between 2-AG concentration and IL-6 in the ADPKD group (Spearman r = 0.31, *p* = 0.02).

To improve the visualization of the relationships among the analyzed variables, a correlation heatmap was generated (Figure 4). The heatmap suggests a pattern of associations between endocannabinoids and renal function parameters. In particular, AEA showed a negative correlation with serum creatinine and a positive correlation with eGFR, suggesting a potential relationship between endocannabinoid levels and kidney function. These relationships were further illustrated using scatter plots. A positive correlation between AEA concentration and eGFR was observed (Figure 5), while AEA showed an inverse relationship with serum creatinine (Figure 6), findings that are consistent with an association between AEA levels and renal function parameters. In addition, a modest but statistically significant positive correlation was found between 2-AG and IL-6 (Figure 7), which may suggest a potential link between endocannabinoid activity and inflammatory processes in ADPKD. Overall, the graphical analyses are consistent with the results of the correlation analysis and highlight potential relationships between components of the endocannabinoid system, kidney function parameters, and inflammatory markers.

### 2.4. Multiple Linear Regression: Effect of 2-AG and IL-6 on AEA Concentration in the ADPKD Group

To identify independent predictors of anandamide concentration, we performed multiple linear regression with stepwise selection. Variables entered into the model were those that were statistically significant in prior analyses. The final model included 2-AG and IL-6 as independent variables. The model was statistically significant (*p* < 0.001) and explained 67% of the variance in AEA concentrations (R^2^ = 0.67).

**Figure 8:**
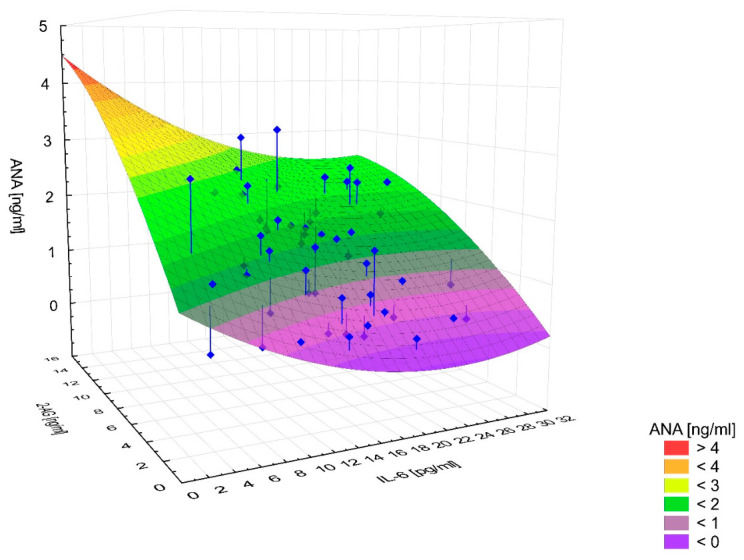
The relationship between the effect of 2-AG and IL-6 concentrations on AEA concentrations in the study group—multiple linear regression model.

2-AG was a strong positive predictor of AEA (β = 0.868; *p* < 0.001), whereas IL-6 showed a significant inverse association with AEA (β = −0.327; *p* = 0.0003) Figure 8. The magnitudes of the standardized regression coefficients indicate that the association between 2-AG and AEA was clearly stronger than that between IL-6 and AEA. Both variables remained significant in the multivariable model, suggesting independent associations with AEA concentration. The regression results are summarized in Table 3.

## 3. Discussion

**Autosomal Dominant Polycystic Kidney Disease as a Systemic Disorder**

Polycystic kidney disease is characterized by the presence of numerous cysts in both kidneys, but cysts also occur in the liver, spleen, and pancreas. The disease is systemic because polycystin proteins are expressed in many tissues throughout the body. Patients often develop a generalized inflammatory state and experience difficulties in glycemic control, which are related to obesity [13,14,32].

In the present study, we compared azotemia parameters between groups. Mean concentrations of creatinine, uric acid, and urea were significantly higher in the ADPKD group than in controls, which is consistent with current knowledge of ADPKD [33]. We also observed higher mean vitamin D levels in the study group and higher mean HGB and RBC values in the control group. These differences most likely reflect extra-renal consequences of the disease. The higher vitamin D levels in individuals with ADPKD likely result from increased supplementation. The elevated cardiovascular risk and disturbances in calcium phosphate metabolism that occur in ADPKD frequently prompt treatment with vitamin D derivatives as part of nephroprotective management [34]. The lower red cell parameters confirmed in our ADPKD cohort compared with controls are consistent with the natural course of the disease [35]. ADPKD is a cause of chronic kidney disease, in which normocytic, normochromic anemia typically occurs. However, according to the current KDIGO 2025 guidelines, individuals with ADPKD, compared with those with other etiologies of CKD, usually have higher hemoglobin levels [36]. This is attributed to local hypoxia, which induces transcription factors leading to erythrocytosis. Nevertheless, as the disease progresses, anemia develops; it is closely associated with CKD progression and constitutes a marker of poor prognosis in ADPKD [35].

The study also provided conclusions regarding the correlation of endocannabinoids with parameters of azotemia and compounds considered uremic toxins. In the study group, in contrast to the control group, a negative correlation was demonstrated between endocannabinoids and urea, creatinine, uric acid, and parathyroid hormone, as well as a positive correlation with GFR. This indicates a link between the endocannabinoid system and the progression of chronic kidney disease in ADPKD. The endocannabinoid system has been implicated not only in diabetic kidney disease but also in non-diabetic nephropathies, where increased expression of the CB1 receptor in the kidneys is observed, correlating with deterioration of renal function and leading to fibrosis [37,38,39,40].

**Association Between the Endocannabinoid System and Inflammation in Polycystic Kidney Disease**

In this study, particular attention was paid to the relationship between the endocannabinoid system and inflammation in polycystic kidney disease. Two proinflammatory cytokines—TNF-α and IL-6 were compared between groups. IL-6 levels were significantly higher in the ADPKD group than in controls. Moreover, IL-6 correlated positively with 2-AG in both groups. No statistically significant correlations were found between TNF-α and AEA or 2-AG in either group.

The experiment also demonstrated a correlation between IL-6 and kidney size in the study group, suggesting a link between inflammation and disease progression. Similarly, two acute-phase proteins, transferrin and ferritin, also correlated with endocannabinoids in the group of patients with diabetes, unlike in the healthy group.

Based on multivariable analysis using a multiple linear regression model, 67% of the variance in AEA concentration was explained by a model including 2-AG and IL-6 as independent variables. 2-AG was a strong positive predictor of AEA, whereas IL-6 showed an independent, significant inverse association with AEA. In standardized terms, the effect of 2-AG was clearly stronger than that of IL-6. These findings may suggest interdependence among components of the endocannabinoid system and a potential link with inflammatory activity.

In the study by J. Klawitter, as in the present work, higher IL-6 levels were observed in patients with ADPKD compared with healthy individuals [41]. These data are consistent with our results and reinforce the important role of inflammation in cyst development in ADPKD and in disease progression [28]. In animal models of polycystic kidney disease, macrophages have been identified as the principal inflammatory cells within the interstitium [26,27,28]. In murine models, elevated levels of proinflammatory cytokines have been demonstrated in the nuclei of cyst lining cells in ADPKD, which aligns with our conclusions regarding IL-6 [42,43].

In a study involving 233 adults with ADPKD, concentrations of several proinflammatory cytokines—including IL-6 and TNF-α—were measured and compared with those of a control group [44]. Patients with ADPKD had markedly higher levels of proinflammatory cytokines. These findings are consistent with our results for IL-6; however, with respect to TNF-α, the present study did not detect statistically significant differences between the ADPKD and control groups.

A number of contemporary studies support a connection between the endocannabinoid system and inflammation [45,46,47,48]. In the work by Berg et al., the effect of anandamide on inflammation in graft versus host disease (GVHD) was examined [45]. Administration of exogenous AEA to murine models produced an anti-inflammatory effect via CB2 receptor activation, reducing lymphocyte migration, decreasing TNF-α, and increasing IL 10 levels, which translated into improved survival in experimental GVHD. Similarly, Borgonetti et al. observed that exogenous cannabinoids suppress inflammatory processes through CB2 activation, including reductions in TNF-α release [46]. In a scientific review on cirrhosis-induced cardiomyopathy, it was noted that endocannabinoids acting via CB1, together with TNF-α and proinflammatory cytokines such as IL-6, contribute to peripheral vasodilation and heightened sympathetic activity, thereby exacerbating myocardial dysfunction [47,48].

The endocannabinoid system functions as a modulatory network, maintaining neural balance and supporting immune responses; as such, it can attenuate inflammation and participate in tissue repair. Current evidence links CB2 receptor activation with anti-inflammatory effects, and its activation may limit tissue injury, including in the kidney [14,49,50,51,52]. CB2 activation inhibits proapoptotic signaling, the release of proinflammatory cytokines, and the formation of inflammatory infiltrates [53,54]. By contrast, CB1 activation promotes renal inflammation via increased expression of oxidative stress markers, indicative of ongoing cellular injury [55]. In a study on diabetic nephropathy, the impact of CB1 receptor activity on disease progression was observed [31]. It was noted that chronic peripheral CB1 receptor blockade or genetic inactivation of this receptor in renal proximal tubule cells (RPTCs) reduces the severity of renal inflammation and mitigates diabetes-induced structural and functional changes in the kidney.

Multiple studies have demonstrated that CB1 and CB2 receptors play important roles in inflammatory and apoptotic signaling pathways in the kidney [53,54,55,56]. Levels of both endocannabinoids and CB2 receptor expression have also been observed under conditions of acute injury, indicating that this receptor participates in endogenous defense mechanisms [57]. Research in liver diseases has confirmed pro- or anti-inflammatory actions of 2-AG, and predominantly anti-inflammatory effects of AEA [21,22]. Interactions with CB1 and CB2 receptors likewise exert opposing effects, acting proinflammatorily (CB1) versus anti-inflammatorily (CB2). In a murine model of diabetic cardiomyopathy, administration of a phytocannabinoid suppressed oxidative stress and inflammation and reduced hyperglycemia through CB2 activation [58]. This study, similarly to ours, supports an association between the endocannabinoid system and inflammation.

Findings regarding TNF-α in the present work are inconclusive. The absence of statistically significant correlations with endocannabinoids and IL-6 may reflect the limited sample size and group heterogeneity with respect to potential comorbidities not captured by the exclusion criteria.

**Role of the Endocannabinoid System in the Pathophysiology of Autosomal Dominant Polycystic Kidney Disease**

In the present study, we compared the concentrations of two endocannabinoids—anandamide and 2-arachidonoylglycerol—between the ADPKD and control groups. Both endocannabinoids were significantly lower on average in the ADPKD group. A significant positive correlation was observed between AEA and 2-AG.

Similar relationships between the endocannabinoid system and ADPKD were reported by Klawitter et al. at the University of Denver [41]. That study included 102 patients with ADPKD and 100 healthy controls and, consistent with our results, found lower levels of AEA and 2-AG in patients with ADPKD compared with healthy individuals [41]. Both studies indicate that dysregulation of the endocannabinoid system is a feature of polycystic kidney disease.

By contrast, studies of obesity-related and metabolic syndrome-associated kidney disease report elevated endocannabinoid levels in both obese animals and humans [32,59]. In another study of hemodialyzed patients, higher serum 2-AG was associated with a lower risk of death in hemodialyzed patients, whereas AEA did not show the same association; notably, the two endocannabinoids appeared to have opposing effects [13,14,60]. In a cohort of 133 young adults, plasma endocannabinoid concentrations were examined in relation to body composition and cardiometabolic risk [61]. Higher plasma endocannabinoid levels were observed in participants with overweight/obesity compared with metabolically healthy individuals, correlating with a worse metabolic profile and increased risk of cardiovascular disease [61,62,63].

In a mouse study, investigators examined whether regulating endocannabinoid levels—particularly AEA—via caloric restriction might confer protection in acute ischemic kidney injury [64]. CB1 receptor inhibition has been shown to lessen cisplatin-induced AKI, suggesting that CB1 blockade may be beneficial in kidney and other inflammation driven diseases [55]. Conversely, other studies have reported that activation of CB1 and CB2 can protect against ischemic AKI by increasing renal blood flow and vasodilation [53,54,65].

Moreover, in another study, ischemic AKI was associated with a marked increase in 2-AG [56].

Regulation of many physiological functions depends not only on the concentrations of endogenous endocannabinoids but also on engagement of specific receptors, whose tissue distributions differ. This differential localization translates into distinct—and often opposing—effects. Further studies are required to fully elucidate the role of the endocannabinoid system in the body, including in the kidney. Clarifying the contribution of the endocannabinoid system to kidney diseases is important given the growing body of evidence linking endocannabinoid system (ECS) dysregulation with proteinuria, AKI, diabetic nephropathy, and renal fibrosis [13,14,39,65,66,67]. Our results likewise indicate that endocannabinoid imbalance occurs in polycystic kidney disease.

### Study Limitations

The study has several limitations. First, due to the exploratory nature and the number of tested correlations, no correction for multiple comparisons was applied, which may increase the risk of type I error. Second, the relatively small sample size may limit statistical power. Finally, the cross-sectional design precludes causal inference.

## 4. Materials and Methods

### 4.1. Characteristics of the Study and Control Groups

The study enrolled 105 individuals under the care of the Department of Nephrology, Transplantology and Internal Medicine, Pomeranian Medical University in Szczecin, Poland. This study was designed as a prospective observational study involving patients with autosomal dominant polycystic kidney disease (ADPKD) and a control group of healthy individuals. The study group comprised patients with autosomal dominant polycystic kidney disease diagnosed according to Ravine’s criteria, supplemented by a positive family (genetic) history, at CKD stages 3–5 as defined by KDIGO guidelines. The study group included 60 participants (29 men and 31 women). The control group consisted of 45 healthy volunteers without a diagnosis of ADPKD (19 men and 26 women). There was no statistically significant difference in age between the study and control groups.

Exclusion criteria for both groups included: diabetes mellitus, active malignancy, coexisting active inflammatory disease, uncontrolled hypothyroidism, current smoking/tobacco use, hematologic diseases, autoimmune diseases, and the use of lipid lowering, immunosuppressive, or antidiabetic medications.

All patients and healthy volunteers provided written informed consent. The study was approved by the Bioethics Committee of the Pomeranian Medical University (approval No.: KB−0012/24/14).

### 4.2. Data Collection

A single peripheral venous blood sample with a total volume of 5.3 mL was collected from each participant (both ADPKD and control). AEA and 2-AG were measured in EDTA plasma. Blood sampling was performed after an overnight fast (12–14 h). Participants were instructed to abstain from alcohol for at least 3 days before sampling and to avoid vigorous physical activity and stress on the day prior to sampling. In both groups, the following laboratory parameters were determined: complete blood count, iron metabolism indices, electrolyte panel, and serum creatinine, urea, and uric acid. In the collected material, endocannabinoids (2-AG and AEA), TNF-α, and interleukin-6 concentrations were also measured. All participants additionally underwent abdominal ultrasonography, during which the longitudinal dimensions of both kidneys were assessed.

#### 4.2.1. Study Procedures

Concentrations of IL-6, TNF-α, AEA, and 2-AG in EDTA plasma were determined by ELISA using commercial kits in accordance with the manufacturers’ instructions (Table 4). From the time of collection into K2EDTA tubes, samples were processed as follows: centrifugation (10 min, 2500 RCF), transfer of plasma into Eppendorf tubes, and storage at −80 °C until analysis. For the final readout, absorbance was measured using an EnVision microplate reader (PerkinElmer, Shelton, Connecticut, USA), and concentrations were calculated from standard curves generated from standards of known concentrations supplied with the kits.

#### 4.2.2. Statistical Analysis

For all parameters, the mean value, standard deviation, median and quartiles—lower and upper—were determined. The significance of differences between age groups and between genders within age groups was assessed using the nonparametric Mann–Whitney U test. Correlations between parameters were analyzed using the Spearman rank correlation test. The threshold of statistical significance was assumed at the level of *p* ≤ 0.05. Variables included in the multiple linear regression model were selected based on prior univariate analyses (Spearman correlations) with *p* < 0.05. A stepwise selection procedure was applied to identify independent predictors of AEA concentration. Due to the exploratory nature of the study and the relatively small sample size, no formal correction for multiple comparisons was applied; this is acknowledged as a limitation. Statistical analysis was performed using the statistical program Statistica PL13 (StatSoft, Krakow, Poland).

## 5. Conclusions

The endocannabinoid system is integral to maintaining physiological homeostasis. Its broad influence on mental and physical health has made it the subject of extensive research. The ECS supports appropriate stress responses, pain reduction, and well-being; it also bolsters immune function, helps regulate metabolism, and exerts neuroprotective effects in the context of neurodegenerative disease. Diet, physical activity, sleep, stress, and lifestyle modulate ECS function. Supporting the ECS—e.g., by consuming omega 3-rich foods or practicing relaxation techniques—may enhance its function and help prevent disease. Endocannabinoids may also serve potentially as biomarkers of severity or decompensation in various chronic diseases.

As demonstrated in this study, patients with ADPKD exhibited lower endocannabinoid concentrations compared with healthy individuals. These findings suggest that restoring appropriate endocannabinoid levels—for instance, by inhibiting their degradation or enhancing their synthesis—could be beneficial and may represent a potential therapeutic target in the treatment of polycystic kidney disease. Furthermore, inflammation appears to be a driver of CKD progression in ADPKD. The endocannabinoid system likely modulates inflammatory processes and may thus participate in disease pathogenesis. Further studies are required to fully delineate the complexity of this regulatory system.

## Figures and Tables

**Table 1:** Statistical parameters of age, blood morphology and biochemical assays in the study and control groups.

Parameter	Study Group (SG) (n = 60, M/F = 29/31)				Control Group (C) (n = 45, M/F = 19/26)				*p*
	Mean ± SD	Me LQ, UQ	Min	Max	Mean ± SD	Me LQ, UQ	Min	Max	
Age [years]	47.43 ± 12.55	47.00 36.00 56.50	26.00	73.00	46.67 ± 9.15	44.00 39.00 54.00	32.00	64.00	0.850
Creatinine [mg/dL]	1.28 ± 0.75	1.02 0.83 1.30	0.68	4.05	0.82 ± 0.12	0.82 0.74 0.87	0.62	1.14	0.000
eGFR [ml/min/1.73]	66.84 ± 26.87	71.76 48.25 83.88	13.82	118.56	86.62 ± 11.13	88.36 76.63 96.46	65.75	104.60	0.000
Urea [mg/dL]	42.97 ± 22.54	36.00 28.00 52.00	14.00	135.00	28.23 ± 8.60	27.50 23.00 32.00	12.00	51.00	0.000
Uric acid [mg/dL]	6.54 ± 2.09	6.30 4.95 7.55	3.10	13.40	5.50 ± 1.41	5.60 4.50 6.70	2.70	7.70	0.018
RBC [T/L]	4.59 ± 0.48	4.60 4.30 4.96	3.46	5.67	4.84 ± 0.49	4.81 4.53 5.15	3.86	6.00	0.018
HGB [mmol/L]	8.42 ± 0.94	8.45 7.80 9.20	6.20	10.30	8.85 ± 0.93	8.90 8.30 9.60	6.60	10.60	0.027
Iron [µg/dL]	102.45 ± 40.62	100.50 76.00 121.00	0,98	233.00	117.88 ± 46.72	115.50 85.00 151.00	23.00	229.00	0.084
PTH [pg/mL]	72.11 ± 64.39	48.30 38.30 69.30	16.40	319.00	60.04 ± 24.39	55.20 41.40 74.30	24.70	126.10	0.418
Calcium ionized [mmol/L]	1.21 ± 0.06	1.22 1.17 1.25	1.10	1.38	1.20 ± 0.073	1.21 1.18 1.24	1.01	1.33	0.740
Phosphorus [mmol/L]	1.08 ± 0.14	1.09 0.97 1.17	0.80	1.55	1.06 ± 0.13	1.07 0.98 1.15	0.74	1.33	0.706
Vitamin D3 [ng/mL]	20.33 ± 8.92	19.60 13.95 24.95	3.00	49.70	15.02 ± 6.18	15.45 10.10 18.50	3.80	28.70	0.003
Ferritin [ng/mL]	115.53 ± 105.94	85.60 46.02 143.45	6.76	560.76	106.70 ± 110.34	73.55 22.64 148.34	2.65	443.02	0.291
Transferrin [g/L]	2.51 ± 0.39	2.53 2.28 2.76	1.56	3.24	2.74 ± 0.33	2.67 2.52 2.88	2.18	3.63	0.008
Length of the right kidney [mm]	164.11 ± 30.80	160.00 140.00 190.00	100.00	220.00	109.86 ± 7.25	110.00 106.00 114.00	96.00	130.00	0.000
Length of the left kidney [mm]	172.29 ± 31.06	170.00 155.00 200.00	120.00	240.00	110.12 ± 22.92	115.00 110.00 120.00	95.00	130.00	0.000

LQ—lower quartile; UQ—upper quartile; SD—standard deviation; Me—median; Min—minimum; Max—maximum; *p*—significance level, the analysis was performed using a U Mann–Whitney test; M/F = male/female; eGFR—estimated Glomerular Filtration Rate; RBC—Red Blood Cells; HGB—Hemoglobin; PTH—Parathyroid hormone.

**Table 2:** The endocannabinoid, TNF-α and interleukin-6 concentrations in the study and control groups.

Parameter	Study Group (SG) (n = 60, M/F = 29/31)				Control Group (C) (n = 45, M/F = 19/26)				*p*
	Mean ± SD	Me LQ, UQ	Min	Max	Mean ± SD	Me LQ, UQ	Min	Max	
AEA [ng/mL]	1.46 ± 0.77	1.47 0.70 1.88	0.32	3.22	3.79 ± 1.64	3.51 2.86 4.51	1.19	8.62	0.000
2-AG [ng/mL]	5.72 ± 3.38	5.36 2.61 8.55	0.63	13.60	14.29 ± 6.95	13.34 8.82 19.88	0.00	28.61	0.000
IL-6 [pg/mL]	15.15 ± 6.93	15.80 10.33 19.97	0.00	28.79	9.65 ± 3.77	9.88 6.50 12.25	1.67	17.33	0.000
TNF-α [pg/mL]	120.08 ± 290.14	34.57 25.29 54.66	12.42	1786.00	90.71 ± 187.19	33.86 24.57 61.00	10.29	973.14	0.872

LQ—lower quartile; UQ—upper quartile; SD—standard deviation; Me—median; Min—minimum; Max—maximum; *p*—significance level, the analysis was performed using a U Mann–Whitney test; M/F = male/female; AEA—Anandamide; 2-AG—2-Arachidonoylglycerol; IL-6—Interleukin-6; TNF-α—Tumor Necrosis Factor-α.

**Table 3:** Analysis of the influence of the tested parameters on the AEA concentration—linear multiple regression model.

Dependent Variable	Independent Variable	β	*p*	R^2^	*p*
ANA	2-AG	+0.868	<0.001	0.67	<0.001
	IL-6	−0.327	0.0003		

AEA—Anandamide; 2-AG—2-Arachidonoylglycerol; IL-6—Interleukin 6.

**Table 4:** ELISA kit data summary.

Set (Name, Category Number, Company)	Material	Standard Curve Range	Sensitivity of the Method
Human IL-6 ELISA Kit, Cat. No. E0090Hu, BT LAB (Bioassay Technology Laboratory, Shanghai, China)	Plasma EDTA	2–600 ng/L	1.03 ng/L
Human TNF-α ELISA Kit, Cat. No. E0082Hu, BT LAB (Bioassay Technology Laboratory)	Plasma EDTA	3–900 ng/L	1.52 ng/L
Human Anandamide (AEA) ELISA Kit, Cat. No. E3875Hu, BT LAB (Bioassay Technology Laboratory)	Plasma EDTA	0.05–20 ng/mL	0.022 ng/mL
Human 2-Arachidonoylglycerol (2-AG) ELISA Kit, Cat. No. BT-E3875Hu, BT LAB (Bioassay Technology Laboratory)	Plasma EDTA	0.05–20 ng/mL	0.022 ng/mL

## Data Availability

The data presented in this study are available on request from the corresponding author. The data are not publicly due to lack of patients’ consent to make their data public.

## Supplementary Materials

The following supporting information can be downloaded at: https://www.mdpi.com/article/10.3390/ijms27094087/s1.

## Abbreviations

The following abbreviations are used in this manuscript:

CKD	Chronic kidney disease
ADPKD	Autosomal dominant polycystic kidney disease
AEA	Anandamide
2-AG	2-arachidonoylglycerol
TNF-α	Tumor necrosis factor α
IL-6	Interleukin 6
EDTA	Ethylenediaminetetraacetic acid
NAPE-PLD	N-acyl-phosphaditylethanolamine-specific phospholipase
DAGLs	Diacylglycerol lipases
FAAH	Fatty acid amide hydrolase
MAGL	Monoacylglycerol lipase
CB1	Cannabinoid receptor 1
CB2	Cannabinoid receptor 2
PKD	Polycystic kidney disease
TNFR1	Tumor Necrosis Factor Receptor 1
TNFR2	Tumor Necrosis Factor Receptor 2
HGB	Hemoglobin
RBC	Red blood cell
GVHD	Graft versus host disease
ECS	Endocannabinoid system
AKI	Acute kidney injury
RCF	Relative centrifugal force
ADHD	Attention-deficit hyperactivity disorder
PTSD	Post-traumatic stress disorder
OMIM	Online Mendelian Inheritance in Man
