# Supplementary material for: Endocannabinoid System-Related Inflammation and Progression of Autosomal Dominant Polycystic Kidney Disease
Source: Int J Mol Sci. 2026 May 2;27(9):4087. doi: 10.3390/ijms27094087 (PMC13163273; doi:10.3390/ijms27094087)
Supplement: Supplementary file 1 [file ijms-27-04087-s001.zip › ijms-4201659-supplementary.pdf]

Supplementary materials for Article

Endocannabinoid system – related inflammation and progression of autosomal dominant polycystic kidney disease

Paulina Simankowicz[1], Barbara Dołęgowska[2], Małgorzata Marchelek- Myśliwiec[1], Katarzyna Dołęgowska[2], Jacek Różański[1], Joanna Stępniewska[1,*]

[1] Department of Nephrology, Transplantology and Internal Medicine, Pomeranian Medical University, Powstancow Wielkopolskich 72, 70–111 Szczecin, Poland
[2] Department of Microbiology, Immunology and Laboratory Medicine, Pomeranian Medical University, Powstancow Wielkopolskich 72, 70–111 Szczecin, Poland
* Correspondence: asia_stepniewska@wp.pl; Tel./Fax: +48–914661196

Table S1. Values of Spearman's rank correlation coefficient between endocannabinoid concentrations and age, biochemical and morphological parameters in the study and control groups.

	Study Group (SG)				Control Group (C)			
**Parameter**	**AEA**	***p*-Value**	**2-AG**	***p*-Value**	**AEA**	***p*-Value**	**2-AG**	***p*-Value**
Age [years]	**-0.39**	0.002	-0.22	0.084	0.19	0.218	0.26	0.090
Creatinine [mg/dl]	**-0.82**	0.000	**-0.67**	0.000	-0.05	0.733	0.15	0.317
eGFR [ml/min/1.73]	**0.68**	0.000	**0.54**	0.000	-0.04	0.776	-0.02	0.872
Urea [mg/dl]	**-0.62**	0.000	**-0.55**	0.000	0.13	0.387	**0.39**	0.010
Uric acid [mg/dl]	**-0.51**	0.000	**-0.34**	0.015	-0.10	0.538	0.18	0.256
RBC [T/l]	0.02	0.897	0.02	0.901	0.14	0.370	0.22	0.139
HGB [mmol/l]	0.08	0.547	0.07	0.569	0.04	0.775	**0.39**	0.008
Iron [μg/dl]	-0.06	0.717	0.14	0.230	0.16	0.240	0.19	0.234
PTH [pg/ml]	**-0.46**	0.000	**-0.37**	0.004	-0.01	0.936	-0.10	0.527
Calcium jonized [mmol/l]	-0.13	0.371	-0.16	0.271	**-0.35**	0.028	-0.02	0.901
Phosphorus [mmol/l]	-0.01	0.922	-0.09	0.481	0.15	0.356	-0.01	0.948
Vitamin D3 [ng/ml]	-0.08	0.561	0.15	0.268	-0.03	0.854	0.08	0.633
IL-6 [pg/ml]	-0.04	0.765	**0.34**	0.008	0.24	0.119	**0.62**	0.000
TNF-α [pg/ml]	-0.07	0.584	0.05	0.708	0.08	0.620	0.22	0.154
Ferritin [ng/ml]	**-0.38**	0.004	-0.15	0.259	0.00	0.993	**0.55**	0.000
Transferrin [g/l]	**0.29**	0.031	0.20	0.132	0.06	0.687	**-0.33**	0.035
AEA [ng/ml]			**0.77**	0.000			0.24	0.119
2-AG [ng/ml]	**0.77**	0.000			0.24	0.119		

p – significance level; eGFR - estimated Glomerular Filtration Rate; RBC - Red Blood Cells; HGB – Hemoglobin; PTH - Parathyroid hormone; IL-6 - Interleukin 6; TNF-α - Tumor Necrosis Factor – α; AEA - Anandamide; 2-AG – 2-Arachidonoylglycerol

Table S2. Values of Spearman's rank correlation coefficient between IL-6 and TNF-α concentrations and age, biochemical and morphological parameters in the study and control groups.

	**Study Group (SG)**				**Control Group (C)**			
**Parameter**	**IL-6**	***p*-Value**	**TNF-α**	***p*-Value**	**IL-6**	***p*-Value**	**TNF-α**	***p*-Value**
Age [years]	0.09	0.502	0.04	0.784	0.17	0.258	0.17	0.257
Creatinine [mg/dl]	0.10	0.485	0.17	0.202	0.22	0.148	0.01	0.972
eGFR [ml/min/1.73]	-0.12	0.383	-0.20	0.135	-0.09	0.576	-0.13	0.397
Urea [mg/dl]	0.06	0.674	0.11	0.433	**0.38**	0.010	0.23	0.125
Uric acid [mg/dl]	0.14	0.321	0.12	0.403	0.21	0.182	0.13	0.426
Length of the right kidney [mm]	**0.33**	0.014	-0.05	0.736	**0.38**	0.014	0.14	0.360
Length of the left kidney [mm]	**0.27**	0.047	0.16	0.237	0.18	0.258	0.17	0.292
RBC [T/l]	-0.04	0.747	-	-	0.29	0.053	-	-
HGB [mmol/l]	-0.11	0.404	-	-	**0.43**	0.003	-	-
Iron [µg/dl]	-0.20	0.125	-0.09	0.494	0.22	0.170	-0.14	0.373
PTH [pg/ml]	**0.34**	0.009	0.07	0.618	-0.18	0.254	0.09	0.556
Calcium jonized [mmol/l]	-0.13	0.357	-0.02	0.884	-0.10	0.547	**-0.53**	0.000
Phosphorus [mmol/l]	0.03	0.793	0.11	0.409	0.13	0.433	0.14	0.389
Vitamin D3 [ng/ml]	**0.27**	0.037	**0.43**	0.000	0.11	0.504	-0.18	0.246
IL-6 [pg/ml]	-	-	0.09	0.490	-	-	0.19	0.216
TNF-α [pg/ml]	0.09	0.490	-	-	0.19	0.216	-	-
Ferritin [ng/ml]	0.20	0.146	-0.08	0.574	**0.61**	0.000	0.05	0.751
Transferrin [g/l]	-0.12	0.340	**0.36**	0.008	**-0.62**	0.000	-0.10	0.534
AEA [ng/ml]	-0.02	0.907	-0.07	0.584	0.13	0.377	0.08	0.620
2-AG [ng/ml]	**0.31**	0.017	0.05	0.708	**0.52**	0.000	0.22	0.154

p – significance level; eGFR - estimated Glomerular Filtration Rate; RBC - Red Blood Cells; HGB – Hemoglobin; PTH - Parathyroid hormone; AEA - Anandamide; 2-AG – 2-Arachidonoylglycerol; IL-6 - Interleukin 6; TNF-α - Tumor Necrosis Factor – α
